# Editorial: Cardiovascular adaptation to extreme environment

**DOI:** 10.3389/fcvm.2022.995297

**Published:** 2022-09-30

**Authors:** Alessandro Pingitore, Francesca Mastorci, Marco Laurino, Claudio Marabotti, Cristina Vassalle

**Affiliations:** ^1^Clinical Physiology Institute, Council National Research, Pisa, Italy; ^2^Fondazione G. Monasterio, Regione Toscana, CNR, Pisa, Italy

**Keywords:** cardiovascular adaptation, extreme condition, hypobaria, hyperbaria, cardioprotection

Extreme physiological conditions can be environmental, such as hypobaric (space or altitude) and hyperbaric (diving) or situational, like strenuous exercise. As shown in Figure 1, all these conditions are discussed in this Research Topic on Cardiovascular Adaptation to Extreme Conditions.

**Figure 1:**
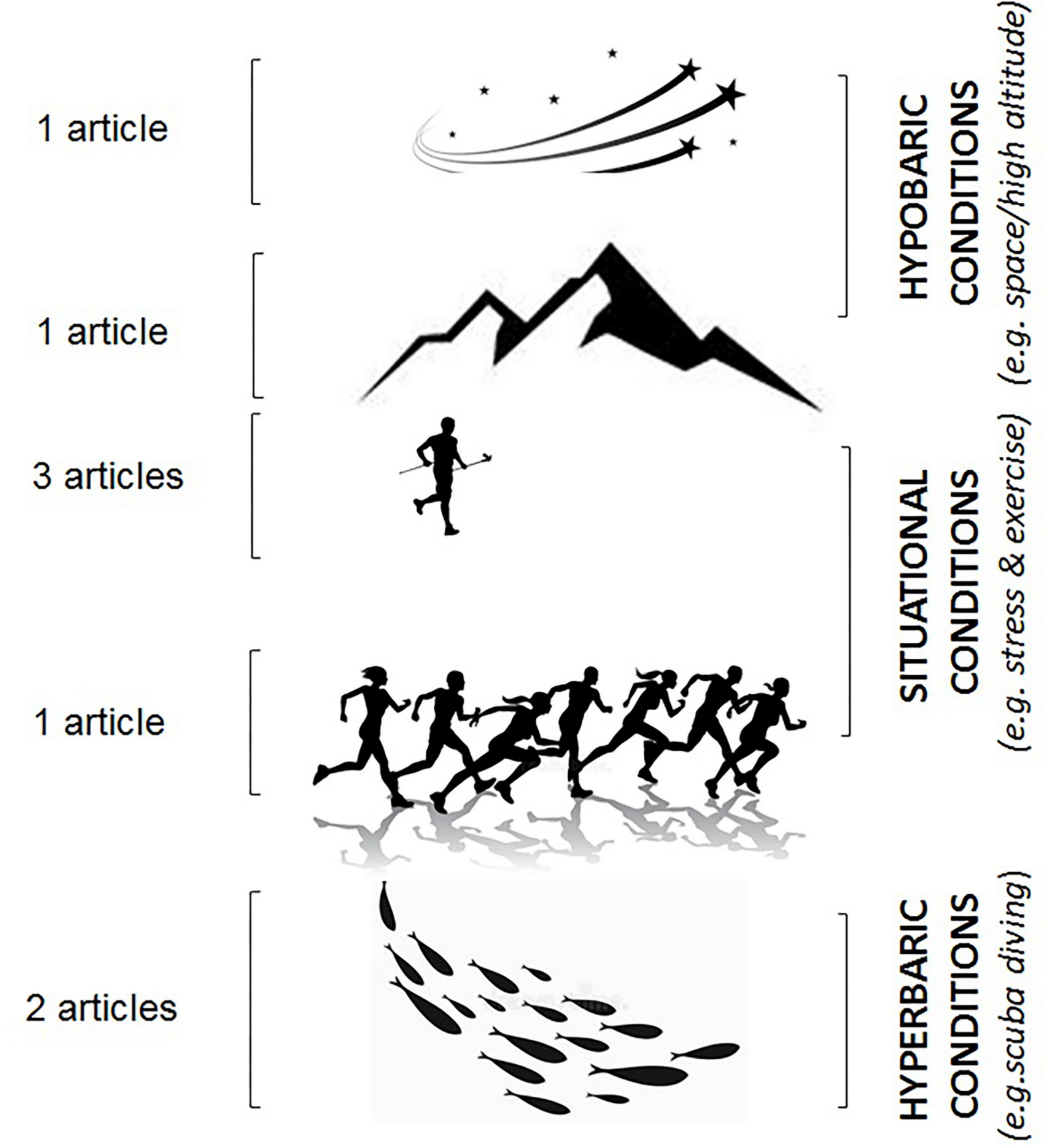
Contributions to the special issue according to hypobaric (space or altitude) and hyperbaric (diving) or situational extreme physiological conditions.

For the hypobaric section, one contribution by Rai et al. focused on monitoring health due to permanence in space; by the evaluation of small nucleolar RNA (snoRNA), a type of short non-coding RNA (60–300 nucleotides), involved in cardiometabolic pathophysiology and cancer. Results showed changes of snoRNAs in peripheral blood mononuclear cells and plasma exosomes in astronauts after a relatively short (median 12 days long) shuttle mission, warning about the significance of the potential translational value of snoRNAs as biomarkers to predict astronauts' health and/or disease.

The article of He et al. discussed the role of neutrophil to lymphocyte ratio, a widely studied index of inflammation in recent years, which is possibly simple to calculate and a cheap biomarker to assess cardiovascular risk in populations living at high altitudes.

Three articles combined the challenging response to high altitude with the demanding effort due to ultramarathon, two focused on biochemical markers, and one on echocardiographic measurements. Specifically, the two manuscripts by Le Goff et al. and Le Goff et al. evaluated the trends of biomarkers in cardiac fibrosis, remodeling, and inflammation in athletes participating in the Ultra-Trail du Mont-Blanc (105 km, total elevation gain: 5,600 m) and in the Tor des Geants^®^ (330 km, +24,000 m in the Valley of Aosta, Italy). In the first manuscript, the evaluated biomarkers increased transiently, most of them returning to baseline levels 7 days after the race, suggesting more of an adaptative response than a real cardiovascular injury. In their second contribution, the authors observed biphasic kinetics of cardiac fibrosis, with a relative recovery during the second half of the event, highlighting that this type of exercise may be less deleterious than shorter and more intense endurance events, and these markers of cardiac fibrosis might be measured and correlated to cardiac magnetic resonance results to assess myocardial fibrosis. Frequently, in ultramarathon running, Wolff et al. assessed exercise-induced cardiac fatigue (EICF) using Doppler echocardiography and myocardial deformation analysis (i.e., global longitudinal strain obtained through speckle tracking echocardiography), as a reduction of >5% of either left ventricular global longitudinal strain or right ventricular free wall strain. Results showed that endurance sports held at mild-to-moderate altitude (1,800–3,200 m) seem to induce EICF in a considerable proportion of recreational athletes, with the decrease in right ventricle functions more accentuated among older subjects. However, training status, or other factors (e.g., race time or dehydration level), did not appear to be associated with EICF. In the context of endurance sports, Venturini and Giallaura discussed the main determinants of the marathon athlete performance to overcome the barrier of 2 h, yet to be broken, with a special focus on environmental (e.g., topography and pollution) and biomechanical factors (e.g., sheltered position, shoe technology), as well as individual characteristics (e.g., nationality, genetics, and sex).

Over the past few decades, scuba diving has become a highly popular and widespread discipline, which, however, places the body under considerable stress, which may vary according to the frequency and intensity of exercise, the duration and load of training, and environmental conditions. Two manuscripts focused on this topic. The article by Dumic et al. evaluated different cardiac and inflammation biomarkers (copeptin; immunoglobulins A, G, and M; complement components C3 and C4; and differential blood count variables, including neutrophil-to-lymphocyte ratio) in recreational SCUBA divers, evidencing the progressive development of a beneficial anti-inflammatory status, which might contribute to cardioprotection and confer other multiple health benefits.

Instead, Mahendiran et al. reported a rare case of spontaneous coronary artery dissection in a 65-year-old female diver, which raises the suspicion of a relationship between physical stress associated with scuba diving and the event, bringing attention to this possible complication in female and older subjects.

In general, the data from these studies show that extreme environments, hyperbarism, and hypobarism, such as extreme conditions, like strenuous and prolonged exercise, have an impact on the cardiovascular system, which reacts through adaptive or homeostatic responses to preserve and protect itself. This response is immediate, transient, and reversible. A testimony to this is Duric's study, which highlights the activation of an adaptive and cardiovascular protective mechanism in SCUBA diving. It can, therefore, be concluded that the adaptive response to an extreme environment or condition by normal subjects is similar to a disease state. However, the substantial difference is that these homeostatic/adaptive mechanisms are transient; i.e., in healthy subjects, they are activated and deactivated, resolving with a return to normal, whereas they are continuously activated in the disease condition. The continuous activation of these systems makes them less efficient over time and, above all, causes a swift of their effect from adaptive to toxic for organs and tissues, favoring the progression of the disease. Thus, the study of athletes, i.e., super healthy subjects, allows us not only to deepen our physiological knowledge of the limits of the human body but also view the improving performance, as well as gain a better understanding of the physiopathological mechanisms activating disease conditions.

## Author contributions

AP and CV wrote the manuscript. FM, ML, and CM reviewed the manuscript. All authors contributed to the article and approved the submitted version.

## Conflict of interest

The authors declare that the research was conducted in the absence of any commercial or financial relationships that could be construed as a potential conflict of interest.

## Publisher's note

All claims expressed in this article are solely those of the authors and do not necessarily represent those of their affiliated organizations, or those of the publisher, the editors and the reviewers. Any product that may be evaluated in this article, or claim that may be made by its manufacturer, is not guaranteed or endorsed by the publisher.

